# Supplementary material for: FZD10 Carried by Exosomes Sustains Cancer Cell Proliferation
Source: Cells. 2019 Jul 25;8(8):777. doi: 10.3390/cells8080777 (PMC6721576; doi:10.3390/cells8080777)
Supplement: Supplementary file 1 [file cells-08-00777-s001.pdf]

FZD10 Carried by Exosomes Sustains Cancer Cell Proliferation

Maria Principia Scavo [1,*], Nicoletta Depalo [2], Federica Rizzi [2], Chiara Ingrosso [2], Elisabetta Fanizza [2,3], Annarita Chieti [1], Caterina Messa [4], Nunzio Denora [2,5], Valentino Laquintana [5], Marinella Striccoli [2], Maria Lucia Curri [2,3] and Gianluigi Giannelli [1,6,*]

1 Personalized Medicine Laboratory, National Institute of Gastroenterology "S. De Bellis", Via Turi 27, Castellana Grotte, 70013 Bari, Italy
2 Institute for Chemical and Physical Processes (IPCF)-CNR SS Bari, Via Orabona 4, 70126 Bari, Italy
3 Dipartimento di Chimica, Università degli Studi di Bari Aldo Moro, Via Orabona 4, 70126 Bari, Italy
4 Laboratory of Clinical Biochemistry, National Institute of Gastroenterology "S. De Bellis", Via Turi 27, Castellana Grotte, 70013 Bari, Italy
5 Dipartimento di Farmacia, Scienze del Farmaco, Università degli Studi di Bari Aldo Moro, Via Orabona 4, 70126 Bari, Italy
6 National Institute of Gastroenterology "S. De Bellis", Scientific Direction, Via Turi 27, Castellana Grotte 70013 Bari, Italy
* Correspondence: maria.scavo@irccsdebellis.it (M.P.S.); gianluigi.giannelli@irccsdebellis.it (G.G.); Tel.: +39-080-4994697 (M.P.S.)

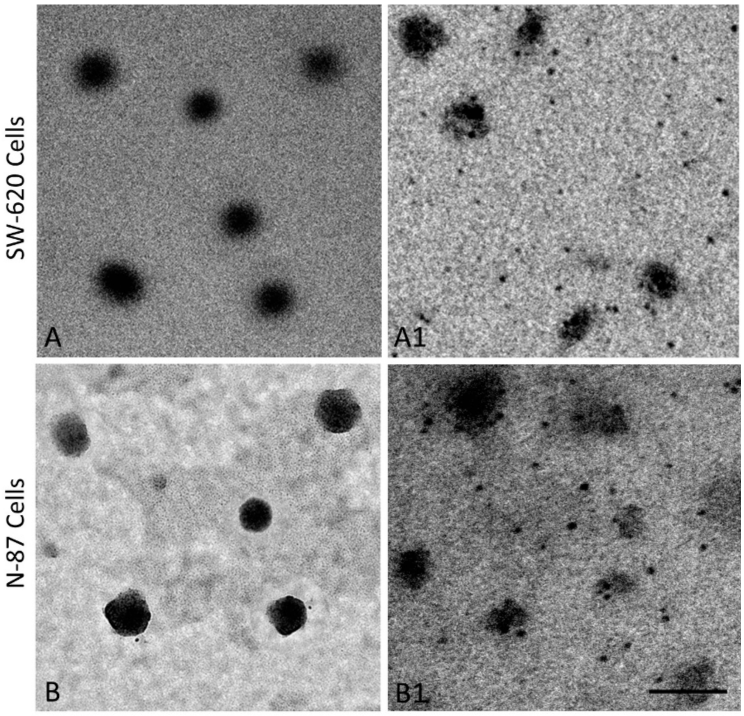

Figure S 1. Representative TEM micrographs obtained with positive staining of exosomes freshly extracted from culture medium of SW-620 (A) and N-87 (B) cells and of as synthesized Au NPs after treatment with FZD10 antibody for 16 hours, with BSA for 1 hour and then with exosomes extracted from SW-620 (A1) or N-87 (B1) cells for 15 minutes. Scale bar 100 nm.

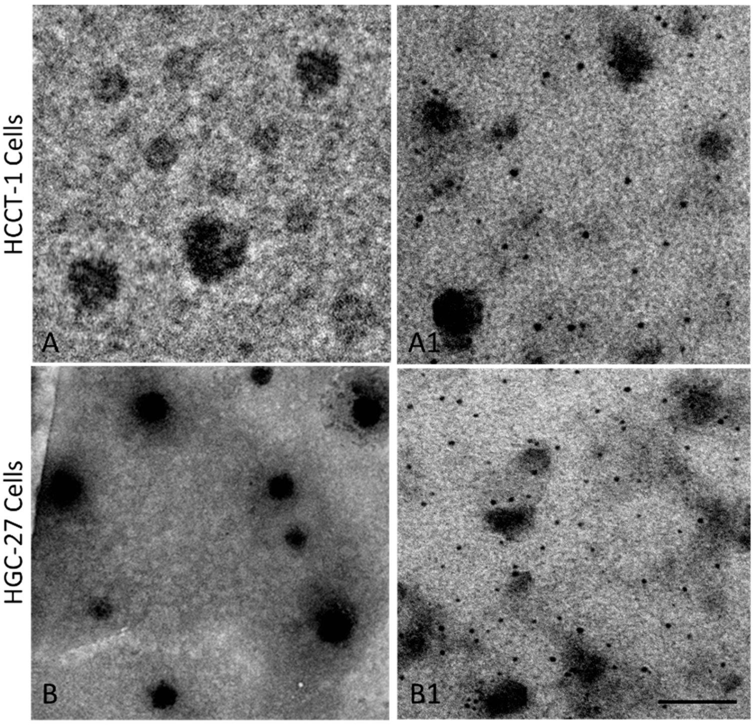

Figure S 2 Representative TEM micrographs obtained with positive staining of exosomes freshly extracted from culture medium of HCCT-1 (A) and HGC-27 (B) cells and of as synthesized Au NPs after treatment with FZD10 antibody for 16 hours, with BSA for 1 hour and then with exosomes extracted from HCCT-1 (A1) or HGC-27 (B1) cells for 15 minutes. Scale bar 100 nm.
